# Five-years post commercial approval monitoring of eucalyptus H421

**DOI:** 10.3389/fbioe.2023.1257576

**Published:** 2023-10-03

**Authors:** Ana Cristina Pinheiro, Anselmo Azevedo dos Santos, Dror Avisar, José Mateus Gonsalves, Maria Paula Galan, Miron Abramson, Noga Barimboim, Othon Abrahão, Rodrigo Neves Graça, Thaís Regina Drezza, William Silva

**Affiliations:** ^1^ Suzano S/A (FuturaGene), Itapetininga, Brazil; ^2^ FuturaGene Israel Ltd. (R&D), Rehovot, Israel; ^3^ DBNBC Brazil Ltda., São Paulo, Brazil; ^4^ W J Silva Consultoria Agrícola S/C LTDA, Jardinópolis, Brazil

**Keywords:** GMO, eucalyptus, biosafety, H421, Cel1

## Abstract

Eucalyptus comprises the largest planted area of cultivated production forest in Brazil. Genetic modification of eucalyptus can provide additional characteristics for increasing productivity, protecting plant yield, and potentially altering fiber for various industrial uses. With this objective, a transgenic eucalyptus variety, event H421, received regulatory approval for commercial release after 6 years of approved risk assessment studies by the Brazilian National Technical Biosafety Commission (CTNBio) in 2015, becoming the first approved genetically modified (GM) eucalyptus in the world. GM event H421 enables increased plant biomass accumulation through overexpression of the *Arabidopsis* 1,4-β-endoglucanase Cel1, which remodels the xyloglucan–cellulose matrix of the cell wall during development to promote cell expansion and growth. As required, in that time, by the current normative from CTNBio, a post-commercial release monitoring plan for H421 was submitted, incorporating general surveillance for five consecutive years with the submission of annual reports. The monitoring plan was conducted on fields of H421 progenies, with conventional clones as comparators, cultivated in representative regions where eucalyptus is cultivated in the states of São Paulo, Bahia, and Maranhão, representing Southeast, Northeast, and Northern Brazil. Over the course of the five-year general surveillance monitoring plan for the approved GM eucalyptus H421, no adverse effect that could impact the biosafety of the commercially approved event was identified. Additionally, the GM eucalyptus exhibited behavior highly consistent with that of conventional commercial clones. Therefore, there was no need for an extra risk assessment study of a case-specific monitoring plan. The results show the importance of continuously updating the regulation norms of governmental agencies to align with scientific advances.

## 1 Introduction

Eucalyptus covers the largest planted area of cultivated production forest in Brazil. The current area of 7.5 million hectares represents approximately 75% of the entire planted forest area in the country (IBGE, 2020; IBA, 2021). The rapid growth, adaptability to a wide range of environments, and high productivity of eucalyptus enables a short crop rotation and offers great potential to meet growing world demand for wood fiber and its derivatives in a sustained and renewable manner whilst avoiding extraction from critical natural forests and preventing irreversible deforestation. Genetic modification of eucalyptus to enhance productivity and to protect trees from pests, pathogens, and herbicide drift offers an additional resource for complementing traditional tree breeding programs. Genetic modification can additionally contribute to the development of eucalyptus trees possessing improved resilience to challenging climate conditions, including drought and heat stresses.

H421 marked a pioneering milestone as the world’s first genetically modified (GM) eucalyptus that has been approved for operational deployment. This unique eucalyptus incorporates a plant gene responsible for regulating the synthesis of endoglucanases, a family of glycoside hydrolases involved in cell wall development and remodeling during growth. These enzymes hydrolyze non-crystalline cellulose and xyloglucans, loosening the cell wall matrix and enabling cell wall plasticity and expansion. In *Arabidopsis*, the endo-(1-4)-β-glucanase Cel1 accumulates in rapidly expanding young tissues and is important for cell elongation (Carpita and Gibeaut, 1993; Shani and Shoseyov, 2006). Research indicates that endoglucanases play a pivotal role in the disentanglement of non-crystalline glucan chains linked to xyloglucans. This enzymatic activity reduces crosslinks, consequently enhancing the flexibility of the cell wall and facilitating the ongoing process of deposition (Shani et al., 2004; Park et al., 2004).

The GM eucalyptus, designated H421, was developed in 2000 via *Agrobacterium tumefaciens*-mediated transformation. This method was used to incorporate the *Arabidopsis* gene *cel1* (1,4-β-endoglucanase) into a hybrid of *Eucalyptus grandis* X *E. urophylla*. H421, which resulted from this transformation process, exhibits enhanced productivity in terms of wood volume per planted area, surpassing that of the corresponding non-GM conventional genotype SP530.

From 2006 to 2015, regulated field trials were conducted to systematically assess biosafety aspects related to H421 across diverse eucalyptus cultivation regions in Brazil to get commercial approval. These trials, encompassing the states of Bahia (S:17°34′26"/W: 39°53′04″), Maranhão (S:06°23′30"/W: 47°06′54″), and São Paulo (S:24°09′37"/W:49°13′09″and S:23°29′40"/W:48°35′38″), aimed to comprehensively assess potential risks to human and animal health, as well as environmental impacts. Rigorous experimental designs and comparable statistical analysis methods were employed to ensure the robustness of the assessments.

It is essential to address concerns and controversies about GM plants due to their putative potential implications for the environment and human health, as well as their socioeconomic implications. Several research studies have demonstrated the significance of this topic (Castaldini et al., 2005; Stone, 2011). Potential environmental impacts and the need for comprehensive biosafety assessments are considered by Brazilian regulation through CTNBio (National Technical Biosafety Commission) and were applied to GM eucalyptus H421. The concerns surrounding GM plants encompass environmental, health, and socioeconomic dimensions, as well as potential impacts on soil microbiota and non-target organisms. To address these concerns, rigorous biosafety analyses and ongoing research are essential, ensuring that GM plants are developed and deployed in a manner that minimizes risks and maximizes benefits.

Both field and laboratory trials were conducted on GM eucalyptus H421, comparing it with its corresponding conventional clone SP530. The objective was to demonstrate the likeness of the agronomic, physiological, morphological, and reproductive traits of H421 to those of the control. Additionally, these trials aimed to assess the chemical composition of leaf and root tissues and pollen, ensuring a comprehensive analysis.

Bioinformatic analysis revealed that the Cel1 and NPTII proteins are not allergenic or toxic. Phenotypic evaluations and ecological interactions indicated that eucalyptus H421 closely resembles its conventional counterpart SP530, thus lacking attributes that pose a heightened risk of invasiveness or dominance within natural habitats. Laboratory and field studies across various edaphoclimatic regions demonstrated comparable seed germination and volunteer plant establishment between clone H421 and the conventional control. Furthermore, pollen grain dimension and viability exhibited no disparities between H421 and the control SP530. No differences were observed between H421 and its conventional counterpart in laboratory-based comparative studies involving non-target organisms such as the fish *Danio rerio*, the invertebrate aquatic *Daphnia similis*, the soil decompositor *Eisenia foetida*, and three beneficial insect species, *Apis mellifera*, *Melipona* sp. and *Scaptotrigona* sp.

CTNBio is the regulatory agency responsible for controlling all the activities of research, development, transport, evaluation, and commercial approval of genetically modified organisms (GMOs) in Brazil. This commission publishes norms governing biosafety procedures for all work on plants, microorganisms, animals, and their derivatives.

Following a comprehensive series of biosafety assessments encompassing laboratory, greenhouses, and field studies of the GM eucalyptus conducted in accordance with the Brazilian Biosafety Law and CTNBio regulations, conclusive evidence emerged affirming that H421 lacks the potential to induce environmental degradation or unfavorable impacts on human and animal health. Consequently, regulatory clearance for GM eucalyptus H421 was sanctioned in 2015.

As stipulated by CTNBio Normative Resolution No. 5 of 2008, a formal request for commercial approval of a GMO in Brazil requires the submission of a post-commercial release monitoring plan. The primary objective of this plan is to meticulously monitor the potential impacts of extensive planting of the GM event and its derivatives, both on the environment and on human and animal health. During this period, adherence to internationally recognized scientific methodologies and appropriately designed experiments was mandated for the monitoring process. In 2011, Normative Resolution No. 9 introduced alternative approaches for the post-release monitoring plan: general surveillance and a case-specific monitoring scheme. General surveillance includes reviewing pertinent technical documents to assess technology utilization, utilizing accessible and appropriate communication channels, administering user questionnaires concerning technology application, and scrutinizing summaries and references if unexpected GM-related biosafety concerns arise in scientific articles published in peer-reviewed journals or in reports by governmental agencies and official notification systems.

An obligatory step in the event of any observed effects during the general surveillance monitoring involves filing a technical report, which is subsequently analyzed by CTNBio.

If the agency deems the effect potentially adverse and linked to GMO usage, an urgent and specific science-based investigation becomes necessary for agency review. Once confirmed, Normative No. 9 mandates the implementation of a case-specific monitoring plan to run concurrently with the general surveillance monitoring. Should the scientific study conducted under the case-specific monitoring plan reveal that the adverse effect indeed poses a biosafety risk associated with the GMO, CTNBio is then tasked with making a critical decision. This decision can involve either suspending the commercialization of the GMO or canceling the prior approval granted by the agency.

If the agency understands that the effect is potentially adverse and linked to the use of a GMO, an urgent and specific science-based study becomes necessary for CTNBio review. If the scientific study conducted under the case-specific monitoring plan reveals that the adverse effect indeed poses a biosafety risk associated with the GMO, CTNBio is then tasked with making a critical decision. This decision can involve either suspending the commercialization of the GMO or canceling the prior approval granted by the agency.

As event H421 conclusively demonstrated biological safety throughout the regulated phase of the scientific investigations and secured commercial approval from CTNBio, a comprehensive general surveillance monitoring plan was submitted to the agency to align with prevailing legislation. The primary objective was to closely monitor the plant characteristics of the GM eucalyptus within cultivated fields across diverse locations where eucalyptus is cultivated in Brazil. This monitoring scheme aimed to identify any unforeseen environmental or human and animal health risks arising from event H421.

Subsequent to CTNBio’s approval, the monitoring plan was implemented over a continuous span of 5 years, starting in 2016. Annual reports were compiled and subsequently approved by the regulatory agency, fulfilling all the requisite regulatory stages for the commercial release of the GM event.

It is important to emphasize that an important advancement was introduced in the latest iteration of CTNBio Normative Resolution No. 32 in 2021 regarding the commercial release of GM events in Brazil. GM products that exhibit negligible effects during the risk assessment stage can be exempted from presenting a post-marketing monitoring plan. This is a progressive step undertaken by the regulatory agency, a testament to the validation of the biosafety evaluation process. This advancement is underpinned by the comprehensive implementation of several post-marketing monitoring plans spanning various crops, species, and traits over the initial 2 decades of the biosafety framework establishment in Brazil.

## 2 Materials and methods

Normative Resolution No. 9, the monitoring process in effect at the time, was based on two main procedures for the detection and evaluation of any effect not foreseen during the risk analysis stage: technical forms/questionnaires and quantitative parameters of forest behavior. Published data related to the GM eucalyptus were also analyzed. During the general surveillance monitoring period, any new signs related to the biosafety of event H421 in cultivated fields or labs not seen before would lead researchers to conduct specific, science-based risk assessment trials to evaluate any putative adverse effects in a case-specific monitoring plan.

The general surveillance monitoring plan, filed with and approved by CTNBio, involved the commercially approved GM eucalyptus H421, its progenies, and conventional eucalyptus clones. BA9318, AO562 and SP5727, respectively, in experimental fields of Bahia, Maranhão and São Paulo.

Post-approval, planting of eucalyptus H421 occurred in specific areas in different eucalyptus-growing regions in Brazil. Planting was scaled up gradually, taking into consideration biosecurity aspects as follow:

Monitoring and post-approval surveillance: Monitoring the performance and safety of GM crops in larger stands under field conditions is crucial. This includes tracking any unanticipated effects on the environment, human health, and silviculture practices.

The first annual report of monitoring post commercial approval of eucalyptus H421 was sent to CTNBio in 2017 and thereafter annually during a period of 5-years. In 2021, a compiled report including data of 5-years of monitoring was sent to regulatory agency confirming the biosafety data that no adverse effect was observed. It means that the data observed during post commercial approval corroborate with previous biosafety data in terms of: Environmental impact assessment (EIA): Evaluating the potential ecological consequences of introducing GM plants into a specific environment. Assessing the impacts on non-target organisms, long-term effects on ecosystems, and potential for gene flow to wild relatives. In Brazil, where no species are sexually compatible with eucalyptus, the assessment of gene flow to wild relatives is not applicable.

Allergenicity: Evaluating whether these changes could lead to increased allergenicity is a critical aspect of biosafety.

Toxicity: Assessing the potential for GM crops to produce toxic substances or unintended byproducts is essential. This includes evaluating the levels of naturally occurring toxins in GM plants.

Food and feed safety: Ensuring the safety of GM plants and their derived products for human and animal consumption is a fundamental concern. This involves assessing nutritional composition, allergenic potential, and potential adverse health effects. However, it is important to note that this evaluation is not applicable to eucalyptus as it is neither used as a feedstock nor for human consumption.

Socioeconomic considerations: Evaluating the impact of GM crops on local communities, farmers, and silviculture practices, also including issues related to access to the technology. It is noteworthy that Suzano has publicly committed to providing its technology to any private grower interested in partnering with the company.

Regulations and compliance: Compliance with national and international regulations and guidelines related to GM plants is essential. These regulations often require comprehensive safety assessments and risk management plans.

### 2.1 Technical forms/questionnaires

The technical questionnaires were compiled annually by field technicians working with the transgenic fields, reporting any differences observed between GM eucalyptus H421 and the conventional controls evaluated in regions where eucalyptus is cultivated in Brazil with different ecosystems, soil types, and forest management practices.

The research team prepared a clear and concise questionnaire document prior to conducting the field visits and administering the questionnaires. This document included introductory information about the objectives of the study and a brief overview of the GM eucalyptus under evaluation. The technical questionnaire was designed to gather valuable insights from specialists and technicians visiting the field trials. It consisted of structured questions covering various aspects of the GM eucalyptus and its interaction with the environment, always comparing the GM eucalyptus to the conventional local clones. Specialists and technicians were scheduled for field visits to the eucalyptus trial sites. During these visits, they had the opportunity to closely observe the eucalyptus plants and their surroundings.

The questionnaire was divided into several categories to cover different aspect, including questions related to the growth patterns, health indicators, and overall condition of the GM eucalyptus. Questions addressed any perceived environmental impacts, including interactions with local flora and fauna. Some questions addressed compliance with biosafety regulations and guidelines. One specific question invited respondents to share any exceptional or unexpected observations. As specialists and technicians responded to the questionnaire, the research team recorded their answers diligently. This process ensured that all responses were accurately documented for later analysis. The findings from the technical questionnaires were interpreted to draw meaningful conclusions about the performance, biosafety, and environmental impact of the GM eucalyptus.

The questionnaire covered the following topics:

• Personal identification and work region (respondents were asked to provide their personal identification details and specify the region where they conducted their work); experience with GM eucalyptus H421 (time of the respondents’ experience in working with GM eucalyptus H421).
• Frequency of visits to the field areas (frequency of their visits to the field trial areas where the GM eucalyptus was cultivated).
• Field observations with focus on different aspects such as soil conditions (respondents were asked to describe and assess the prevailing soil conditions in the study areas); eucalyptus management practices (questions explored the techniques and practices related to the management of eucalyptus in the field); insect-pest species and/or pathogens (information on the number of insect-pest species present and their respective populations); weed competition (respondents provided insights into the presence and impact of weed competition in the eucalyptus trial sites)
• These observations consistently involved comparisons between H421 and conventional clones.

The questionnaire consistently asked respondents to compare various aspects between GM eucalyptus H421 and the conventional eucalyptus clones.

### 2.2 Parameters of forest behavior

Comparative observations of fields planted with H421 and conventional clones adapted to each planting region were collected in fields with similar management practices in São Paulo, Bahia, and Maranhão, from planting time in 2016 until the end of the fifth year of monitoring in 2021. Parameters such as survival rate after planting, uniformity among plants of the same genotype, and phytosanitary conditions were evaluated.

In each area, the performance of H421 and conventional clones was compared using a production unit score (NUP):

$$NUP=\frac{\left(\left(0,4\;uniformity+0,3\;survival+0,3\;phytosanitary\right)+\left(\frac{\left(0,4\;uniformity\;X\;0,3\;survival\;X\;0,3\;phytosanitary\right)}{\left(3,6\right)}\right)\right)}{2}.$$

Survival was evaluated by counting planting failures among the sampled plants (calculating the percentage of failures and assigning a survival score from 0 to 10). For example, if the survival was 100%, the score was 10. If the survival was below 90%, the score was 0.

The uniformity of the stand was evaluated by measuring the height of the sampled plants of the same genotype (calculating the coefficient of variation (CV) of the height and assigning a uniformity score from 0 to 10). If the CV was smaller than 10%, the score was 10, but if the CV was higher than 40%, the score was 0.

The phytosanitary condition was defined by identifying damage to plants by diseases, pests, mechanical damage, or phytotoxicity. Numerous native and invasive pathogens and pests threaten eucalyptus trees in Brazil. The primary objective of this analysis was to contrast the natural occurrence of pathogens or pest infestations within the GM plot against the plots containing conventional clones. In the first year after planting, the presence of weed competition in the area was also verified. However, after the trees grew taller than 20 m, this parameter became insignificant and was disregarded in the evaluations after the third year of planting.

Annually, 125 trees of each genotype, out of approximately 1,200 trees, were randomly selected and measured within each area to assess various parameters. This analysis comprehensive involved determining the survival rate (number of dead trees), analyzing the degree of uniformity (measuring tree height and quantifying the consistency among them), and evaluating the phytosanitary condition (grade for weeds and diseases affecting the quality of the tree).

### 2.3 Analysis of published data

Scientific articles published on the H421 event (or other GM eucalyptus) during the monitoring period were attached to the annual reports.

## 3 Results

The comprehensive set of observations gathered through visual inspections and rigorous data collection, along with insights derived from questionnaires completed during the five consecutive years of the general surveillance monitoring plan in the test fields, indicated from a biosafety perspective that H421 and the conventional operational clones behaved similarly in the three locations, with no evidence of any biosafety issues of concern requiring continuation of the general surveillance monitoring.

### 3.1 Technical forms/questionnaires

During the 5 years of monitoring, 240 questionnaires were filled out by forest specialists and forest technicians working in the fields. The number of questionnaires analyzed during the entire monitoring period and the distribution by region/area and year is shown in Table 1.

**TABLE 1:** Questionnaires completed by the technicians and specialists during the monitoring period showing no differences observed related to biosafety.

Location area	Planted area	Total # of questionaries applied						Significant observation
		First year (2016)	Second year (2017)	Third year (2018)	Fourth year (2019)	Fifth year (2020)	5 years (sum)	
Itabatã/BA	4.7	8	13	21	15	14	71	No
Açailândia/MA	3.0	14	25	26	18	16	99	
Lençois Paulista/SP	4.4	16	17	13	12	12	70	
All areas	12.1	38	55	60	45	42	240	

The questionnaires completed by the field technicians demonstrated that all the biosafety observations were similar in both the H421 eucalyptus and the conventional clones across different biomes of the geographical regions where eucalyptus is cultivated in Brazil.

### 3.2 Parameters of forest behavior

Eucalyptus H421 and conventional clones showed similar growth behavior during the evaluation period in all the trial areas of São Paulo, Bahia, and Maranhão. The monitoring program showed no differences in silvicultural aspects between GM eucalyptus H421 and its conventional clones (Table 2).

**TABLE 2:** Results of evaluations (production unit score: NUP) obtained in the initial period and from the first to the fifth year of monitoring of the genetically modified eucalyptus, event H421, compared with the control (conventional clone) in three areas: SP, São Paulo; BA, Bahia; and MA, Maranhão.

Area	Material	Production unit score: NUP						Mean (5 years)
		Initial	First year	Second year	Third year	Fourth year	Fifth year	
SP	H421	8.1	9.7	9.6	7.9	8	7.9	8.5 (±0.8)
	Control	7.1	8.8	8.7	9.3	9.4	9.5	8.8 (±0.8)
BA	H421	9.8	9.8	9.7	9.8	9.9	9.9	9.8 (±0.1)
	Control	9.8	9.9	9.4	9.6	9.8	9.4	9.7 (±0.2)
MA	H421	9.6	9.8	9.8	9.4	9.1	8.8	9.4 (±0.4)
	Control	9.8	9.4	10	9.4	8.1	7.3	9 (±1.0)
General mean	H421	9.2 (±0.8)	9.8 (±0.0)	9.7 (±0.1)	9 (±0.8)	9 (±0.8)	8.9 (±0.8)	9.3 (±0.4)
	Control	8.9 (±1.3)	9.4 (±0.4)	9.4 (±0.5)	9.4 (±0.1)	9.1 (±0.7)	8.7 (±1.0)	9.2 (±0.3)

### 3.3 Publication analysis

Several peer-reviewed papers related to the GM eucalyptus published after commercial approval and during the monitoring period were considered relevant:

These publicly available, peer-reviewed publications on GM eucalyptus (Fernandes, 2014; Lucas et al., 2016; Silva et al., 2017; Silva and Abrahão, 2020) indicate that H421 is similar to conventional clones in biosafety aspects, thus posing no increased threat to human and animal health or the environment compared to conventional eucalyptus.

## 4 Discussion

Achieving maximum production per unit of land area is the primary objective of breeding programs for plant species of economic significance. The genetic gain per year is an issue to be considered in the case of tree species that have longer growth cycles. In this context, genetic modification techniques were explored as an additional tool to complement conventional plant breeding methods, aiming to enhance the yield of eucalyptus clones per hectare. Systematic, regulated risk assessment studies were conducted across diverse eucalyptus cultivation regions in Brazil. After extensive research conducted across laboratories, greenhouses, and field trials spanning several years, the GM eucalyptus variant, H421, has emerged as an exemplar of this potential with substantial potential for revolutionizing the forestry industry. These findings were supported by systematic and regulated risk assessment studies conducted across diverse eucalyptus cultivation regions in Brazil. Consequently, the Brazilian National Technical Biosafety Commission (CTNBio) endorsed the commercial release of H421 in 2015, marking it as the world’s inaugural approved GM eucalyptus. This approval carries profound implications for the forestry sector and raises important questions concerning its commercial application.

Throughout the post-commercial release monitoring period, no adverse effects associated with biosafety risks were observed. This observation corroborates the validity of the regulatory data required for the submission and approval of GM event H421. Given the absence of adverse effects noted during the surveillance monitoring plan, event H421 was not required to undergo a case-specific monitoring plan. The data, gathered through the regulated laboratory, greenhouses, and field studies, approved by CTNBio in 2015, and derived from the overall surveillance monitoring plan, validate the biosafety of event H421. These observations establish that eucalyptus H421 possesses a safety profile equivalent to that of conventional clones. No evidence of adverse effects on the environment or human and animal health resulting from the commercial release and large-scale cultivation of the GM eucalyptus H421 was discovered. This information was officially published in the Official Gazette No. 233 on 13 December 2021. The outcomes from the comprehensive monitoring plan for the eucalyptus H421 confirm the report issued by CTNBio during the approval process for the event’s commercial release. Each annual report was evaluated by different member-rapporteurs, confirming that no biosafety risks were identified and that no further H421-related studies were needed, and CTNBio approved all annual monitoring plan reports.

In Brazil, eucalyptus plantations have been established on degraded pasture or land previously deforested. Eucalyptus event H421, known for its high wood yield, has the potential to act as a protective barrier against erosion and to enhance carbon sequestration. This potential contribution supports the advancement of more sustainable practices in eucalyptus plantations. Biotechnology plays a crucial role in the tree breeding programs of species with long cultivation cycles, such as eucalyptus. Genetic modification represents a powerful tool that can synergize with classical breeding programs by incorporating genes from a variety of plant species or even other organisms. This method can significantly bolster yield gains throughout the course of breeding cycles. Thus, the synergy between genetic modification and classical breeding programs accelerates the development of improved eucalyptus varieties. The consistent absence of adverse effects discovered through the 5-year general monitoring of commercially approved eucalyptus H421 indicates the necessity for a continuous review of regulatory norms to align them with scientific advancements in a more streamlined manner. In line with this, CTNBio published Normative Resolution No. 32 in 2021, introducing new adjustments that offer guidance for petitions seeking commercial release. Approved GMOs belonging to Risk Class 1, along with their derivatives, will be exempt from monitoring when the risk assessment conducted by the Internal Biosafety Commission (Institution Commission) and validated by CTNBio does not identify significant risk for the cultivation of the new GM event. Therefore, following the implementation of the mentioned Normative Resolution, event H421 should no longer require monitoring assessments for post-commercial approval.

The approval of H421 for commercial release brings about a new era in eucalyptus cultivation, with clear benefits and obstacles. H421’s potential to increase wood yield per hectare has profound implications. It extends beyond mere economic advantages, potentially serving as a safeguard against soil erosion and as a mechanism for enhanced carbon sequestration, a crucial alignment with global sustainability objectives. However, the commercialization of GM plants, particularly in tree species such as eucalyptus, introduces regulatory, societal, and environmental challenges that require meticulous consideration.

To realize the full potential of H421 while addressing these complex challenges, a comprehensive strategy for its responsible commercialization is imperative. This strategy requires harmonization of regulatory norms with scientific advancements to streamline the approval process, foster innovation, and expedite the introduction of H421 into commercial forestry practices. One essential step toward facilitating the pathway for commercial application is the recent introduction of Normative Resolution No. 32 by CTNBio in 2021. This synchronization of regulation with scientific evidence is key, as it not only ensures timely commercialization but also strengthens the biosafety of H421. It eliminates redundant monitoring efforts for well-characterized organisms, which is essential to optimizing resource allocation and reducing regulatory burden.

The commercial application of H421 extends beyond regulatory measures. It necessitates a cooperative effort among stakeholders, including the forestry industry, regulatory bodies, and the community. Robust stewardship programs must be implemented to monitor and manage the deployment of H421 in the field. This involves rigorous compliance with recommended practices, ongoing assessment of environmental impacts, and proactive mitigation of unforeseen consequences. Furthermore, public engagement and transparent communication are critical elements in achieving a socially accepted and sustainable commercial application of H421. Building public trust and addressing concerns through informative and open dialogues will be instrumental in the acceptance and support of this innovative approach to eucalyptus cultivation.

## 5 Conclusion

Developing a GM tree such as eucalyptus H421 is a time-consuming process, spanning from gene discovery to approval from the regulatory agencies. The combination of biotechnology and classical plant breeding holds the potential to expedite the development of more productive and sustainable GM eucalyptus varieties. Regulatory laws and guidelines, originally applied uniformly to all organisms, evolve to integrate new scientific methods and data. These adaptations, which streamline biosafety evaluations and monitoring processes for GMOs that have proven to be low risk through rigorous safety studies, are needed to address time constraints. The current scenario shows the need for continuous alignment among stakeholders—regulators, product proprietors, and biosafety agencies—to keep up with the rapid pace of the approval of GM events.

This synchronization is essential to facilitate the timely commercialization of safer and more sustainable products, such as eucalyptus H421, while ensuring responsible, vigilant supervision, and stewardship. Sustained cooperation among all parties is critical for optimizing policies and protocols in the evaluation and monitoring of GMOs, ultimately benefiting society and the environment.

## Data Availability

The raw data supporting the conclusion of this article will be made available by the authors, without undue reservation.
